# Integration of Rabbit Adipose Derived Mesenchymal Stem Cells to Hydroxyapatite Burr Hole Button Device for Bone Interface Regeneration

**DOI:** 10.1155/2016/1067857

**Published:** 2016-01-05

**Authors:** Viswanathan Gayathri, Varma Harikrishnan, Parayanthala Valappil Mohanan

**Affiliations:** Biomedical Technology Wing, Sree Chitra Tirunal Institute for Medical Sciences and Technology, Poojappura, Thiruvananthapuram, Kerala 695 012, India

## Abstract

Adipose Derived Mesenchymal Stem Cells, multipotent stem cells isolated from adipose tissue, present close resemblance to the natural* in vivo* milieu and microenvironment of bone tissue and hence widely used for in bone tissue engineering applications. The present study evaluates the compatibility of tissue engineered hydroxyapatite burr hole button device (HAP-BHB) seeded with Rabbit Adipose Derived Mesenchymal Stem Cells (ADMSCs). Cytotoxicity, oxidative stress response, apoptotic behavior, attachment, and adherence of adipose MSC seeded on the device were evaluated by scanning electron and confocal microscopy. The results of the MTT (3-(4,5-dimethylthiazol)-2,5-diphenyl tetrazolium bromide) assay indicated that powdered device material was noncytotoxic up to 0.5 g/mL on cultured cells. It was also observed that oxidative stress related reactive oxygen species production and apoptosis on cell seeded device were similar to those of control (cells alone) except in 3-day period which showed increased reactive oxygen species generation. Further scanning electron and confocal microscopy indicated a uniform attachment of cells and viability up to 200 *μ*m deep inside the device, respectively. Based on the results, it can be concluded that the in-house developed HAP-BHB device seeded with ADMSCs is nontoxic/safe compatible device for biomedical application and an attractive tissue engineered device for calvarial defect regeneration.

## 1. Introduction

Burr hole cranial neurosurgical procedures often retain cosmetically abhorrent puckered scars on the scalp over burr hole sites [1]. Reconstructive surgeries using burr hole button fabricated from biomimetic bone substitutes are currently being used to renovate the residual depression on the scalp over the burr hole [2]. Even though the conventional method seems to be promising, strategies on improvising the local integration and healing process are always demanding [3].

The recent advancement in tissue engineering and regeneration technologies has paved the way for a conceptual shift in the otherwise conventional practices in orthopedic and reconstructive surgery of using prosthesis and other implantable devices [4]. Use of biodegradable scaffolds integrated with biological cells, particularly stem cells, has shown to promote the repair and/or regeneration of tissues plus excellent integration to the surrounding tissues. The stem cell based therapy aims at integrating endogenous mesenchymal stem/progenitor cells (MSCs) with appropriate three-dimensional scaffolds along with other biochemical cues to enhance the healing process particularly the local integration of the construct to the surrounding tissues [5].

Adipose Derived Mesenchymal Stem Cells (ADMSCs) have become a focus of research due to their multipotential properties, high responsiveness to distinct environmental cues, and ease of isolation [6]. Adipose tissue can be easily harvested from subcutaneous tissue through percutaneous or minima open aspiration techniques [7]. The use of ADMSCs to treat calvarial defects is well documented [8]. Implanted poly(lactic-coglycolic acid) scaffolds seeded with ADMSCs promoted complete bone bridging in 12 weeks in a rat model of calvarial defects [9]. The osteogenic potential of adipose stem cells on scaffolds has been examined in* in vitro* cell cultures and* in vivo* animal models, for successful healing and accelerated regeneration process in calvarial and other bone defects [10]. So, it is hypothesized that osteogenesis/osteoinductive factors produced by the adipose stem cells along with a biomimetic bone substitute such as hydroxy apatite would aid in engineering an ideal bone substitute to heal burr hole or other calvarial defects.

As in any tissue engineering procedures, the possible clinical outcome depends on the cell survival in 3D scaffolds which is again defined by various factors such as adequate seeding density, cell attachment and proliferation, and diffusion of nutrients and oxygen supply. Also the immediate cell death after implantation due to hypoxia and other stress factors is generally thought to be the cause of failure of bone tissue engineering strategies. This could be taken by culturing the stem cells on the porous scaffold for a short period ensuring sufficient cell growth prior to implantation onto the defect site instead of the on the spot repair as seen in conventional treatment modalities. The size of the device will be manipulated depending on the size of the defect.

Hence, the objective of the present study is to evaluate the interaction and cytocompatibility of adipose mesenchymal derived stem cells (ADMSCs) and in-house developed hydroxyapatite burr hole device with potential implication on treating burr holes and other calvarial defects using stem cell based tissue engineering strategies.

## 2. Materials and Methods

### 2.1. Preparation of Hydroxyapatite Burr Hole Button Device (HAP-BHB)

**Figure 1:**
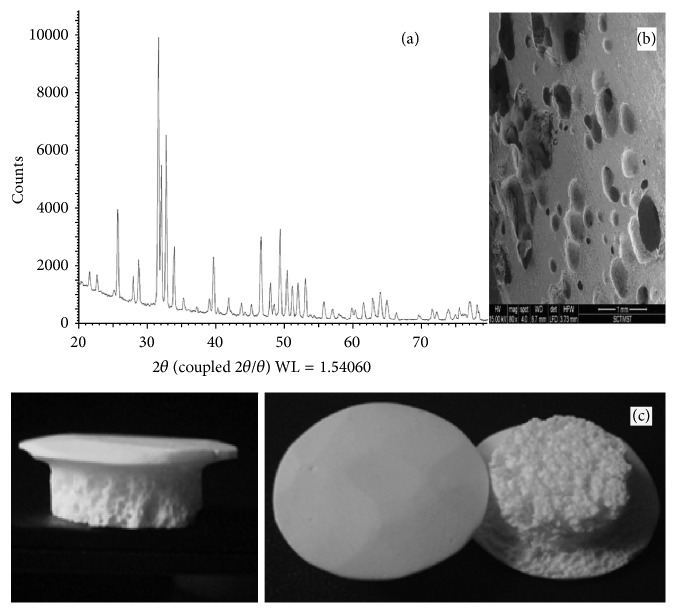
(a) Physicochemical characterization of HA burr hole buttons device using XRD spectrum of the powdered device material with the standard pattern of hydroxyapatite (PDF 9-432) superposed. (b) SEM micrograph of HA Burr Hole Buttons showing porosity distribution. (c) HA-Burr Hole buttons.

The HAP-BHB device (Figure 1(c)) [will be referred to as “device” in the text] developed by the Biomedical Technology Wing of the Sree Chitra Tirunal Institute for Medical Sciences and Technology (Patent India 495/CHE/2006 dated 20/3/2006) consisted of two parts; a column made of porous hydroxyapatite (HA) which “dips” into the burr hole defect (pore size range: 100–300 mm), and a dome which “sits” on the surrounding bone made of dense HA (pore size less than 100 mm). It has a convex surface and could be contoured with mechanical drills. The thickness of the dome was 2 mm at the centre and 1 mm at the periphery. The column of the device was 3-4 mm long which was designed for easy insertion into the burr hole with the dome of the device being as superficial cover. The button could be implanted at any burr hole site irrespective of the location. Precipitation method was employed for the preparation of hydroxyapatite, consisting of calcium and phosphate salts, and the precipitate was washed thoroughly to remove ions adsorbed on the surface. The precipitate was later gel casted, dried slowly, and shaped according to requirements. Sintering was done at 1200°C to get the final ceramic. The Ca/P ratio was 1.67 mimicking mammalian bone. The implants were subjected to standard sterilization techniques [1].

### 2.2. Isolation, Culture, and Characterization of Rabbit Adipose Derived Mesenchymal Stem Cells (ADMSCs) Using Specific Surface Marker (CD+ 90)

Mesenchymal stem cells were isolated from rabbit's adipose tissue. The adipose tissues which were collected from the cadaver as per the approval from Institutional Animal Ethics Committee (CPCSEA, Committee for the Purpose of Control and Supervision on Experiments on Animals, India) and in accordance with the approved Institutional protocol. ADMSCs isolation was done as described elsewhere [11]. Briefly, 10 g of tissue was incubated with 30 mL of 1.5 mg/mL collagenase 1 (Invitrogen) at 37°C with continuous shaking for 30–45 min. Enzymatic dissociation of tissue was stopped by the addition of double the volume of serum-containing medium and the resultant suspension was passed through 180 *μ*m nylon mesh (Millipore). Cells were washed by centrifugation. The cell pellet was resuspended in complete medium consisting of high glucose Dulbecco's Modified Eagle's Medium (DMEM; Gibco), 10% fetal bovine serum (FBS; Gibco), 100 units/mL penicillin, 100 *μ*g/mL streptomycin, and 0.25 *μ*g/mL Fungizone (Antibiotic-Antimycotic (100x) Gibco, USA) solution. The cells were seeded onto a 25-cm^2^ tissue culture polystyrene dish (TCPS) and kept at 37°C under 5% CO_2_. The medium was replenished at 72 h intervals [11]. The medium changes continued until the cells reach approximately 80% confluence. Thereafter, the cells were subjected to trypsinization, 0.25% trypsin-EDTA (Gibco, USA), as described elsewhere [12]. ADMSCs, in passage 2 or 3, were selected for all the* in vitro* experiments. In brief, the plastic-adherent cells after second passage were analyzed with a positive marker (CD+ 90). Finally, the cells were washed and imaged using fluorescent microscope (Leica DMIL, Germany).

### 2.3. Evaluation of Plastic Adherence of ADMSCs by Actin Staining

ADMSCs were grown on TCPS and later stained for F-actin. After incubation for 24 h at 37°C, the cells were washed with PBS and fixed with freshly prepared 4% para formaldehyde for 20 min. Permeabilization of adhered cells was carried out by incubation with 0.1% Triton-X for 2 min at room temperature. Soon after, the cells were incubated in rhodamine phalloidin (Sigma, USA) diluted 1 : 100 in phosphate buffer saline (PBS) for 15 min, following which the cells were washed and treated with Hoechst (Sigma, USA) for staining of nuclei for 5 min. Finally, the cells were washed and imaged using fluorescent microscope (Leica DMIL, Germany).

### 2.4. Assessment of Material Mediated Cytotoxicity of ADMSCs by MTT (3-(4,5-Dimethylthiazol)-2,5-diphenyl tetrazolium bromide) Assay

The cytotoxicity of suspensions of powdered material on ADMSCs cells was assessed by MTT (3-(4,5-dimethylthiazol)-2,5-diphenyl tetrazolium bromide) assay. Briefly, ADMSCs at a density of 20,000–40,000 cells/mL were seeded onto 96-well tissue culture plates and cultured in DMEM with 10% FBS. After 24 h of seeding, the medium was replaced by the medium containing device powdered material at different concentrations (0.0005 g/mL, 0.005 g/mL, 0.05 g/mL, and 0.5 g/mL, resp.) and cultured again for 24 h. Cells supplied with culture media alone served as negative control. After the incubation period, the metabolically active cells were quantified using MTT assay and compared with untreated control. Phenol (1%) served as positive control and ADMSCs alone as negative control. For MTT assay, 10 *μ*L of MTT dye (5 mg/mL in PBS) was added to each well in dark and incubated for 3 h at 37°C. The optical density was measured spectrophotometrically following dissolution in dimethyl sulfoxide (DMSO) at 540 nm (Elx 808iu Ultra Microplate Reader, Bio-Tek Instruments, USA).

### 2.5. Evaluation of Cell Morphology of ADMSC Seeded on the Device (*In Vitro*) Using Scanning Electron Microscopy

ADMSCs were seeded (1 × 10^6^ cells/material) on all sides of preconditioned device and were placed in 35 mm^2^ single well plate (Nunc, Germany) under static condition. The device was coated on all the sides by the ADMSCs in the medium. After being incubated at 37°C for 1 hour to allow cell attachment, DMEM with 10% FBS (Gibco, USA) was added for 24 h. The seeded device was maintained for 3, 7, and 14 days, independently [13]. The ADMSC seeded device was washed with PBS, fixed in 1% glutaraldehyde in Sorensen phosphate buffer for 24 hours and dehydrated in a graded ethanol series and the morphology of adhered and attached ADMSC on the device was visualized by Scanning Electron Microscopy (Hitachi 2500).

### 2.6. Measurement of Oxidative Stress Potential (Reactive Oxygen Species Level)

The generation of ROS was monitored by employing 2,7, Dichloro Dihydro Fluorescein Diacetate (DCF-DA) [Invitrogen, USA]. DCF-DA fluoresces when oxidized by the intracellular ROS generation. Briefly, ADMSCs (20,000–40,000 cells) were preincubated with DCF-DA at a concentration of 100 *μ*M. After washing with serum-free medium, the cells were treated with varying concentrations of powdered device material (0.0005, 0.005, 0.05, and 0.5 g/mL, resp.) for 2 h, at 37°C. Hydrogen peroxide (0.09% H_2_O_2_) treated cells were used as positive control for DCF-DA analysis. Cells were then washed twice in serum-free medium and the resulting fluorescence was quantified using a fluorescence microplate reader with an excitation wavelength of 488 nm and emission wavelength of 53 nm. The values were normalized to the negative control (ADMSCs alone) and respective fold change was calculated.

### 2.7. Evaluation of Live/Dead ADMSC Seeded on the Device Using FDA and PI Staining by Confocal Microscopy

Fluorescence-based live-dead assays were used to evaluate the viability of cells. Simultaneous use of two fluorescent dyes allows a two-color discrimination of the population of live cells from the dead-cell population. Seeded ADMSCs on the device were stained with fluorescein diacetate (FDA) and propidium iodide (PI), which stain viable cells and dead cells, respectively. Briefly, the staining solution (culture medium without fetal calf serum (FCS) 5 mL + FDA (5 mg/mL) 8 *μ*L + PI (2 mg/mL) 50 *μ*L) was added upon the device seeded with ADMSCs and was incubated at room temperature for 4 to 5 minutes in the dark. Later, staining solution was removed and subsequently PBS was added to wash the device seeded with ADMSCs. Samples were analyzed with confocal laser microscopy [14]. Future studies will concentrate to measure the bone regenerating ability of the HAP-BHB and to demonstrate the efficacy of the device.

### 2.8. Flow Cytometric Evaluation of Apoptosis by Annexin V/Propidium Iodide (PI) Staining

Briefly, ADMSCs were seeded on the device for different time periods (3, 7, and 14 days) independently. Later, the device seeded with the ADMSCs were washed with PBS and trypsinized. The cell suspension was then double stained with Annexin V-Alexa Fluor and propidium iodide as per the manufacturer's protocol (Vybrant Apoptosis Assay Kit, Molecular Probes, Invitrogen, USA). The stained cells were then analyzed for apoptosis using BD FACSAria III flow cytometer.

### 2.9. Statistical Analysis

For all experiments, three separate devices were used (*n* = 3). All the measurements were done in triplicate in order to confirm the repeatability. Each parameter was expressed as mean of all values ± standard deviations. One-way analysis of variance (ANOVA) was employed to assess the statistical significance of results. *p* values less than 0.05 were considered significant.

## 3. Results

### 3.1. Physicochemical Characterization of HAP-Burr Hole Buttons

The phase purity of hydroxyapatite is ascertained by XRD compared with powder diffraction data (JCPDS 9-432) standard. The calcium to phosphorus ratio was estimated by EDTA titration and spectrophotometer method and observed as 1.66. The flexural strength is estimated in the three-point bending method and is found to be 139 MPa with a standard deviation 25 (Figure 1(a)). Microstructure study has been carried out on a Hitachi scanning electron microscope. The figure is a SEM micrograph of the interface showing porosity as well as the high sintered characteristics (Figure 1(b)).

### 3.2. Isolation, Culture, and Expansion of ADMSCs and Characterization by CD+ 90 Marker

**Figure 2:**
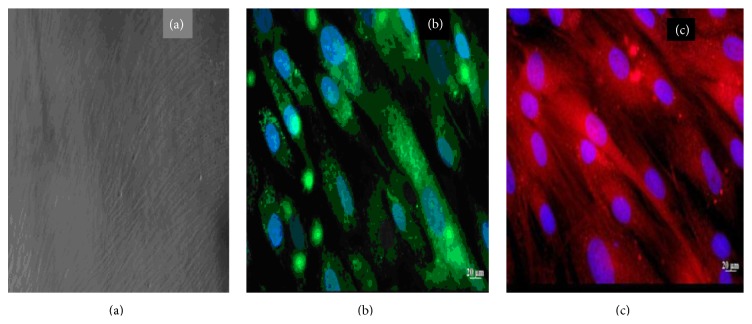
(a) Spindle-shaped morphology of rabbit adipose MSCs. (b) Staining of CD+ 90, specific cell surface marker. (c) Actin staining showing cytoskeletal arrangement.

The ADMSCs were isolated from a heterogeneous population by virtue of their plastic adherence property and were successfully propagated in primary culture and in serial culture on TCPS. They attached in colonies and spread out from individual colonies. The ADMSCs exhibited their characteristic fusiform, spindle-shaped morphology from day 2 (Figure 2(a)). Medium given was DMEM high glucose supplemented with 10% fetal bovine serum. The culture attained 80% confluence by 7 days. Cells of passages 2-3 were used for all the studies. The ADMSCs were characterized by the specific surface marker CD+ 90 staining and viewed under fluorescent microscope (Figure 2(b)).

### 3.3. Evaluation of Cytoskeletal Arrangement and Plastic Adherence Property of ADMSCs by Actin Staining

ADMSCs are polystyrene/plastic adherent cells and are shown to spread quickly upon adhesion with an increase of cytoskeleton-associated actin (F-actin). Thus, the staining of cytoplasmic actin filaments was performed to demonstrate the plastic adherence property of ADMSCs. Fluorescent images of actin staining are shown in (Figure 2(c)).

### 3.4. Determination of Cytotoxicity of ADMSCs by MTT Assay

**Figure 3:**
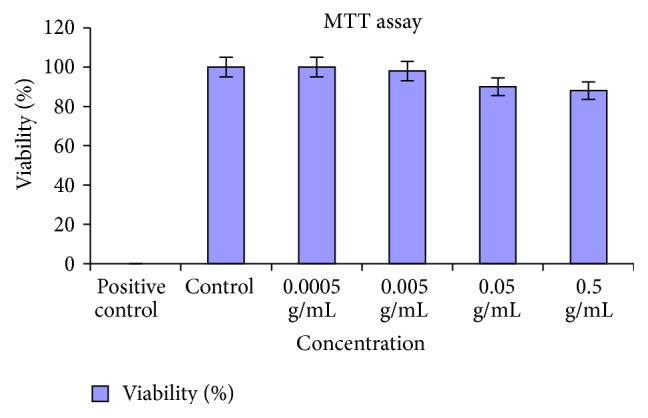
Cell viability by MTT assay. All values are expressed as mean ± Std. deviation (*n* = 6).

Cell viability was quantitatively estimated employing colorimetric MTT assay that detects mitochondrial activity of the cells. It is based on the reduction of the yellow tetrazolium dye 3-(4,5-dimethylthiazol)-2,5-diphenyl tetrazolium bromide (MTT) to a purple water insoluble formazan in cells bearing intact mitochondria and hence reflects the state of cultured cells. Results depicted that percentage viability of ADMSCs when treated with powdered device material (Figure 3) was similar, when compared with negative control (cells alone). 1% phenol served as positive control. There was no significant difference in the values with the increase in concentration powdered device materials up to 0.5 g/mL that was found to be noncytotoxic when compared with control.

### 3.5. Evaluation of Cell Morphology and Attachment of ADMSC Seeded on the Device Using Scanning Electron Microscopy

**Figure 4:**
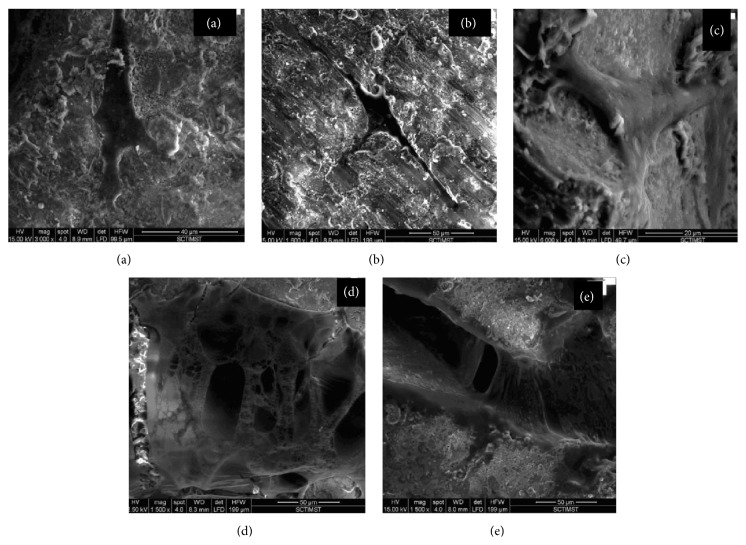
Scanning electron micrographs showing attachment and adhesion of ADMSCs on the device. (a and b) Day 3 attachment of ADMSCs on the device. (c) Day 7 attachment of ADMSCs on the devices showing filopodia formation. (d and e) Day 14 attachment of ADMSCs on the device showing layers of cells (ADMSCs) on the device.

As evident from scanning electron micrographs, ADMSCs attached and adhered on the device from day 3 onwards. Three-day seeded ADMSCs on the device micrographs depicted typical ADMSCs morphology (Figures 4(a) and 4(b)). Eventually, ADMSCs were increased and distributed evenly over the device to give a cell sheet like appearance with filopodia (Figure 4(c)) on day 7. On day 14, ADMSCs formed sheets and layers of cells which were seen on both sides of the device (Figures 4(d) and 4(e)).

### 3.6. Measurement of ROS Generation of ADMSCs Seeded on the Device

**Figure 5:**
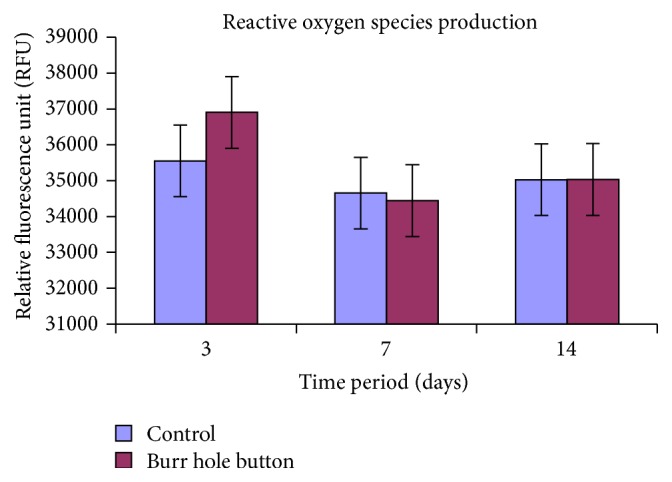
Measurement of the level of reactive oxygen species. All values are expressed as mean ± Std. deviation from three different replicates.

Figure 5 represents the intracellular ROS generation level of ADMSCs after exposure to powdered device material at different time periods (3, 7, and 14 days). The study showed a 10-percent increase in ROS generation on 3-day treated period when compared to control. Subsequently, this increase in ROS production on day 3 was neutralized at 7 and 14 days.

### 3.7. Evaluation of Live/Dead ADMSC Seeded on the Device Using FDA and PI Staining by Confocal Microscopy

**Figure 6:**
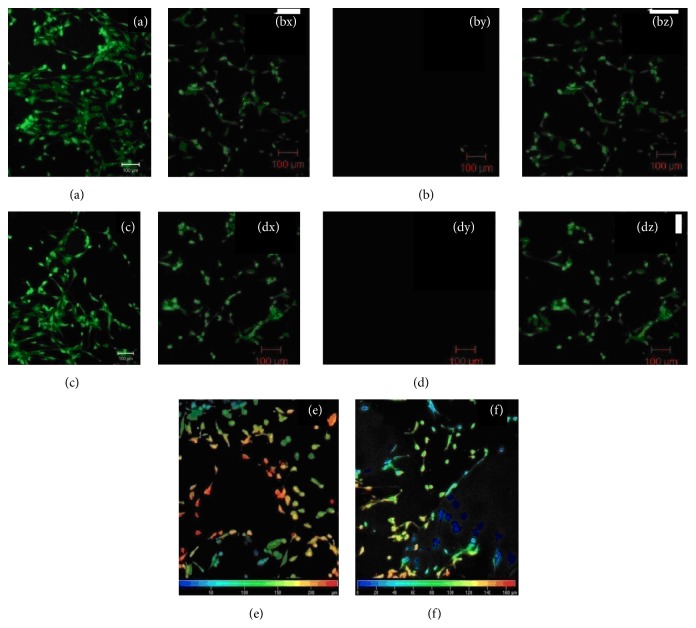
Confocal images showing ADMSCs attachment on device. (a) Porous surface of the material. (bx, by, and bz) Overlay image of live and dead cells on porous surface of the material (c) Dense surface of the material. (dx, dy, and dz) Overlay image of live and dead cells on dense surface of the material (e) Depth code analysis of cell viability on the porous surface. (f) Depth code analysis of cell viability on the dense surface.

Confocal images showed that ADMSCs were significantly attached on porous surface of the device and exhibited viability (Figure 6(a)). Overlay images of porous surface is shown in Figure 6(b). Also, necrotic cells were absent on the porous surface. Similarly, dense surface of the device (Figure 6(c)) showed attachment of ADMSCs.  Figure 6(d) shows overlay image of dense surface. Necrotic cells were very negligible on the dense surface also. Eventually, Figures 6(e) and 6(f) showed the depth code analysis of ADMSCs attachment and adherence on porous and dense surface of the device, respectively. It was noted that viable cells were attached up to 200 *μ*m inside the devices.

### 3.8. Evaluation of Device Seeded with ADMSCs for Apoptosis Using Annexin V by Flow Cytometry

**Figure 7:**
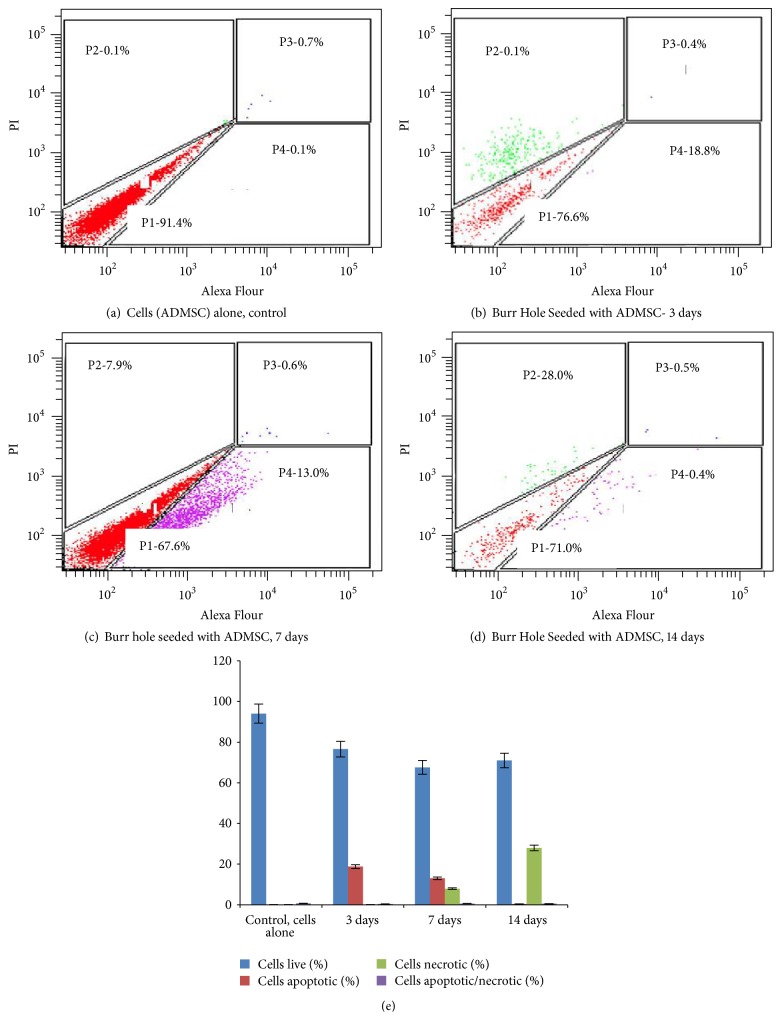
Flow cytometry evaluation for apoptosis of ADMSCs seeded onto the device. Annexin V assay. (a) Unstained ADMSCs control; (b) 3 days; (c) 7 days; (d) 14 days; (e) graphical representation of quantitative data (*n* = 3). All values are expressed as mean ± SD from three different replicates.

Early and late apoptotic cells were detected using Annexin V FITC-propidium iodide kit by flow cytometry analysis (FACS BDAria). The data showed the presence of early apoptotic cells (16%) on the device seeded with ADMSCs on 3 days when compared to control (0.0%); the percentage of late apoptotic cells as well as necrotic cells was found to be negligible. This may be due to the initial stress experienced by the cell on acclimatization to the new substrate. However, following incubation, it was found that the apoptotic cells were not significant when compared with cells (ADMSCs) alone control. The data on day 7 and day 14 could not be correlated as events evaluated were not comparable with the 3rd day data.  Figure 7 depicts graphical representation of percentage live/apoptotic/necrotic cells.

## 4. Discussion

Calvarial and craniotomy defect rebuilding is still a challenge to many tissue engineers. Bone has the extraordinary capacity to heal without scar formation, but this regenerative capacity is impaired in patients with large bone lesions and patients with impaired wound healing process. An ideal bone substitute is one which can repair or replace large bone defects especially calvarial segmented defects. Ideally bone substitute should show (a) biocompatibility, (b) osteoconduction, (c) osteointegration, (d) osteoinduction, (e) osteogenesis, and (f) vascularisation [15]. Available materials for the above-mentioned applications display many of these properties, whereas properties like osteoinduction and vascularisation potential are exceptional in the available therapies. These processes are associated with the presence of growth factors or cells within the scaffold/material/device. Here lies the importance of tissue engineering, which utilizes a 3D porous biomaterial with cells and growth factors to provide structural support after neurosurgery procedures.

With hydroxy apatite being a natural constituent of bone and teeth, most of its applications are focused on the area of bone regeneration and dental restoration. Tissue engineered hydroxyapatites are particularly appealing because of the high biomimetic morphologies for applications such as orthopedic implant coating, bone substitute filler, and burr hole buttons during craniotomy [16].

Cytocompatible evaluation is the preliminary step in evaluating the toxicological behavior of any biomaterial. In this study, the results showed no evident abnormal morphological lesions in the cells (ADMSCs) exposed to device. Subsequently, the viability of ADMSCs obtained from MTT assay at all the exposed concentration of the powdered device material showed no cytotoxicity. Thus, mechanical damage to the cells was not observed.

The duration of culturing of cells on the materials* in vitro* prior to* in vivo* studies is a crucial feature that ensures the success of tissue engineered constructs. Some studies suggested cells to be cultured on the scaffolds for few hours, while, in other systems, the cells were cultured for one or more weeks before implantation [17]. Former approach gives an idea of cell attachment and proliferation time and the latter approach creates an extracellular matrix on the scaffold that provides signals towards the defect site. Several studies with rat bone marrow mesenchymal stem cells established that the bone formation was generated more effectively by culturing cells on the scaffold for a short* in vitro* culture period (days 1–8) [18]. Keeping the above facts in mind, the present study adopted both approaches to view cell attachment and adherence. Therefore, a period of 3, 7, and 14 days was selected to study the attachment and adherence of the cells (ADMSCs) on the device. The performance of cells (morphology, viability and attachment, and adherence) on the scaffolds was examined initially in an* in vitro* cell culture system to ensure that the cells can attach and adhere. Likewise, the SEM images and confocal microscopy depicted the morphology, attachment, proliferation, and viability of ADMSCs on the device, respectively.

The toxic effect of material at cellular level is evident by cellular death. Two distinct pathways adopted for cell death are apoptosis and necrosis. Acute cellular dysfunction in response to severe stress conditions leads to necrosis which is a relatively passive process coupled with rapid cellular depletion. The executor behind this fact is oxidative stress rising due to disturbance in the prooxidative, antioxidative balance resulting in cell damage [19]. Apoptosis is a form of programmed cell death that occurs during several pathological situations and is a complex process characterized by cell shrinkage, chromatin condensation, and formation of blebbing and apoptotic bodies [20]. However, 3-day result showed an increase in ROS level by the ADMSCs seeded on the device. But, this effect was seen neutralized in 7 and 14 days, respectively. Consequently, change in ROS level on the 3rd day could be the consequence of adapting to the new environment which subsequently subsided on further incubation.

Formation of new vasculature on the defect site is an important parameter for osteo induction and for osteogenesis to happen [21]. Various* in vivo* studies show the rapid vasculature with BMSc and ceramics [22]. Hartman et al. in 2004 [23] found that muscle recipient site could favour bone formation in a cell based bone graft substitute compared to subcutaneous recipient site due to high vascularity of the muscle tissue.

Tissue-specific differentiation of MSCs is dependent on the local microenvironment in which these cells reside and their interactions with the host tissue. Rat bone marrow mesenchymal cells covered on nanohydroxyapatite coated on silk fibroin Nano-HAp/silk. Fibroin sheets initiates adhesion and proliferation [24]. Similarly, the observations from the current study suggest enhanced ADMSCs adherence, attachment, and viability on the device which is necessary for the bone regeneration and vasculature. Preliminary cytocompatibility data obtained from this study implies the use of device seeded with ADMSCs for bone tissue engineering applications. The efficacy of the device will be demonstrated on the rat model with a calvarial defect for 1-, 4-, and 12-week period.

## 5. Conclusions

In this study, the interaction and cytocompatibility of adipose mesenchymal derived stem cells (ADMSCs) and in-house developed device are evaluated as a preliminary step in projecting hydroxyapatite burr hole integrated with mesenchymal stem cells to improvise the healing process and local integration of burr hole button in neurosurgery patients. The device is found to be compatible with the stem cells seeded and it can be concluded that the in-house developed HAP-BHB device seeded with ADMSCs are nontoxic/safe compatible device for biomedical application and may be an attractive tissue engineered device for calvarial defect regeneration in future.
